# Medico-Chirurgical College

**Published:** 1883-01-20

**Authors:** F. Ll Sieure Weir

**Affiliations:** Clinical Professor of Dermatology


### MEDICO-CHIRURGICAL COLLEGE.

### SERVICE OF F. LL SIEURE WEIR, M.D.,

Clinical Professor of Dermatology. Reported by Dr. H. B. Nightingale, Chief of Clinic.

### ACNE.

CASE I. The young man before you to-day is an Englishman by birth, 21 years old and unmarried. His occupation is that of a laborer. About 6 years ago his face, which had previously been clear of any eruption, showed here and there small, red pimples, some of which pustulated, others again remaining stationary for a few days and then disappearing. From being a few in number they gradually increased in number until he was 18 years old, at which time he began to shave. Since then the condition now observable has been present, new crops appearing to take the place of those which had run their course. His appetite has always been good; in fact, too good. Bowels inclined to constipation. He sleeps well; has never had gonorrhœa or syphilis. A close inspection of the face shows this eruption to consist of small, elevated points or pimples, which are solid and have a feel not unlike the pustules of smallpox, vivid red in color, and causing some little sense of discomfort by the tension of the epidermis, commencing at or shortly after puberty, with no specific history, an evidently deranged digestion and habitual constipation.

These give us all that is necessary to know about the disease. Masturbation is a very frequent cause of this affection, or, if not a direct cause, largely assists in keeping up the irritation. There is no history of such practice in this case.

He will be ordered 5 gr. doses of lactopeptine 3 times a day, to be followed by a bitter tonic. Also the following:

℞ Ext. cascara sagrada, fld. . . . . . . . . . . . . . . . . . . . . . . . . . f℥ j.

Sig. Gtt. x, in sweetened water, before each meal and at bed-time.

This will have the effect of producing a free evacuation daily, and if he will continue its use for some time his constipation will yield permanently. I know of no drug which relieves habitual constipation so effectually as the cascara sograda. Locally he shall have a soothing ointment of oxide of zinc and cosmoline, with a few drops of carbolic acid. The local treatment is secondary, for upon the relief of the dyspepsia and constipation a marked change will be observed. He will be directed to let the beard grow, thereby removing one potent cause of irritation.

### ERYTHEMA SIMPLEX.

CASE II. A. M., 30 years, Irish, and employed as a cook in one of our hotels. She is constantly bending over a hot range, owing to her occupation, and some weeks ago noticed that the redness of the face, which had previously disappeared after a time, began to remain longer. Presently it became so bad as not to leave at all, and gave rise, in addition, to burning, smarting and more or less itching. The redness, as you have seen, involves pretty much the whole face, not. however, producing a uniform discoloration. The redness is in patches, which coalesce with each other, leaving a ragged margin. By passing the finger over the surface the patches are found to be very slightly raised, and the redness disappears, returning again instantly upon removing the pressure. It is a case of erythema simplex, or superficial inflammation of the skin, from external irritation. In the way of treatment, removal of the exciting cause is the primary object. In all idiopathic cases it is the one thing to do. If we can get the patient's co-operation in this, we will order her a simple oxide of zinc ointment, with possibly a few drops of carbolic acid.

### URTICARIA.

CASE III. H. J., 18 years. Here is a young lady who comes to us suffering from "the hives" Here, upon the forearms, you see these large, red blotches, each with a pale or whitish centre, no regularity as to distribution, but lying close together on some parts of the limb, while upon the hand, considerably removed from the others, are more patches. They are quite prominent, and give rise to a most exasperating sensation of burning and itching. Even now you see she cannot refrain from scratching the parts. Both arms and hands, and the face as well as neck, are involved. Doubtless the body and lower limbs share the affection in turn. There is never any difficulty in recognizing the disease, the wheals are so characteristic. In no other disease do we find wheals appearing suddenly and, after remaining a varying time, disappearing as rapidly and mysteriously as they came. It is a most common difficulty and the diagnosis is easily made out. But *what csuses* it is not always so easy to ascertain. A case of urticaria or nettle rash in which the exciting cause is unknown is one of the most stubborn and unsatisfactory of all, and the doctor to whose lot it falls is apt to become disgusted with the study of dermatology. The exciting causes of urticaria are divisible into three heads: local irritants, a polluted circulation and reflex irritation. Without going deeper into the subject, let me say that the first two causes are easily disposed of, and it now remains to be seen *hcw* reflex irritation is responsible. The patient yesterday morning indulged in fish for breakfast, and in the course of the afternoon felt a burning and smarting upon various parts of the body. It was not severe, however, till night, when she got warm in bed, at which time it became almost unbearable. Once or twice before fish has had the same effect upon her, but not for several years. She is of a nervous temperament, cvidently, and this fact renders it all the more easy for the disease to manifest itself. My assistant tells me that, just before coming into the room, there was no sign of wheals; yet, upon her entrance, I was able to show you some very fine specimens of them. The disease is, I feel convinced, a neurosis, not alone in the case before us, but demonstrably so in every case. The divisions I gave you a few moments ago are made for the sake of convenience only, and if the first two so-called heads be eliminated I think that reflex action can be clearly shown to be the cause of the cutaneous phenomena in every case.

The treatment in the present case shall consist of three compound cathartic pills. Considerable constipation is present, and as no stool has been had since the eating of the fish, it will have the effect of removing the remnants of it and clearing out the alimentary canal. Locally, a lotion, as follows:

℞ Ammoniæ carbonatis.......................... ʒ ss

Plumbi acetatis.............................. ʒ j

Glycerinæ.................................... f ℥ j

Aquæ rosæ.................................... f ℥ v. M.

Sig. Use as a wash, several times daily.

Without doubt our patient will obtain relief by these measures.

### ECZEMA AURIUM.

CASE IV. Willie S., aged two years. The child before you is brought here on account of an eruption which made its appearance in the neighborhood of the ears about two and a half months ago. As you see, not only the cleft behind them, but also the auricles themselves, are involved. The inflammation, however, does not seem to have extended into the auditory canal to any appreciable extent. A hot, red, tense and swollen surface, with a thin, serous fluid constantly bathing the parts; numerous small vesicles, in varying stages of development; a feverish state of the body and constant fretting; a strumous diathesis, poor appetite and costiveness, all of which are present, mark the case unmistakably as one of eczema. The suffix of aurium designates the seat, not that it differs from the same variety in any other portion of the body.

This state of affairs has been going on for a period of two or three months, the symptoms becoming gradually worse until life has become a burden to him, young as he is. If allowed to go on the symptoms would probably not change noticerbly, except the discharge, which would become purulent. Indeed, it is so to a slight extent already. The only wonder is that such has not been the case long ere this. Being a catarrhal inflammation essentially, it is more apt to assert itself in an intractable form when occurring in lymphatic children.

We must try to relieve the little fellow, and to this end will advise the following to be applied to the parts two or three times daily:

℞ Zinci oxidi,

Zinci carbonatis, } ............................aa ʒ ij

Glycerinæ................................ f ℥ iij

Aquæ rosæ......................q. s. ad f ℥ iv. M.

Sig. Shake well before using.

The surface is to be dried by *pressing* gently with a piece of soft muslin. Never *wipe* an inflammation. This lotion should leave a fine powder upon the surface, and by the protection which it affords the parts the air is excluded. He shall have, also:

℞ Potassæ chloratis................................ʒ j.

Fiat. chart. No. xx.

Sig. A powder to be dissolved in water and taken thrice daily.

Were the patient older we could administer cod-liver oil with advantage, but young children do not, in my opinion, assimilate it so well, and, moreover, will not take it. The chlorate of potassa here answers an admirable purpose as an alterative, and in the present case will, I feel sure, fulfill all the indications of the oil. Some time will be required to effect a cure, but I think we may safely predict success in the end. The mother will be directed to bring him back in a week, at which time he will be shown you, and any necessary changes in the treatment effected.

